# Prefrontal theta—gamma transcranial alternating current stimulation improves non-declarative visuomotor learning in older adults

**DOI:** 10.1038/s41598-024-55125-2

**Published:** 2024-02-29

**Authors:** Lukas Diedrich, Hannah I. Kolhoff, Ivan Chakalov, Teodóra Vékony, Dezső Németh, Andrea Antal

**Affiliations:** 1 Department of Neurology, University Medical Center Göttingen, Göttingen, Germany; 2 Department of Anesthesiology, University Medical Center Göttingen, Göttingen, Germany; 3 Centre de Recherche en Neurosciences de Lyon CRNL U1028 UMR5292, INSERM, CNRS, Université Claude Bernard Lyon 1, Bron, France; 4 BML-NAP Research Group, Institute of Psychology, Eötvös Loránd University and Institute of Cognitive Neuroscience and Psychology, Research Centre for Natural Sciences, Budapest, Hungary; 5 Department of Education and Psychology, Faculty of Social Sciences, University of Atlántico Medio, Las Palmas de Gran Canaria, Spain

**Keywords:** Implicit learning, Transcranial alternating current stimulation (tACS), Cross-frequency coupling, Cognition, Non-declarative memory, Aging, Elderly, Neuroscience, Cognitive ageing, Cognitive neuroscience, Learning and memory

## Abstract

The rise in the global population of older adults underscores the significance to investigate age-related cognitive disorders and develop early treatment modalities. Previous research suggests that non-invasive transcranial Alternating Current Stimulation (tACS) can moderately improve cognitive decline in older adults. However, non-declarative cognition has received relatively less attention. This study investigates whether repeated (16-day) bilateral theta—gamma cross-frequency tACS targeting the Dorsolateral Prefrontal Cortex (DLPFC) enhances non-declarative memory. Computerized cognitive training was applied alongside stimulation to control for the state-of-the-brain. The Alternating Serial Reaction Time (ASRT) task was employed to assess non-declarative functions such as visuomotor skill and probabilistic sequence learning. Results from 35 participants aged 55–82 indicated that active tACS led to more substantial improvements in visuomotor skills immediately after treatment, which persisted 3 months later, compared to sham tACS. Treatment benefit was more pronounced in older adults of younger age and those with pre-existing cognitive decline. However, neither intervention group exhibited modulation of probabilistic sequence learning. These results suggest that repeated theta—gamma tACS can selectively improve distinct non-declarative cognitive aspects when targeting the DLPFC. Our findings highlight the therapeutic potential of tACS in addressing deficits in learning and retaining general skills, which could have a positive impact on the quality of life for cognitively impaired older individuals by preserving independence in daily activities.

## Introduction

As the global population continues to age, there is an increasing need to understand and address the cognitive changes associated with human aging. One area of particular interest is the decline in memory functions, which can significantly impact an individual's daily functioning and quality of life^1–3^. For declarative memory, which encompasses conscious recall of facts and events, studies consistently demonstrated a decline with age^4–6^ or in the presence of a cognitive disease such as Mild Cognitive Impairment (MCI) or Alzheimer’s Disease (AD)^7,8^. In contrast, results are controversial with respect to non-declarative memory, which is responsible for implicit learning, skill acquisition, and habit formation^9^. While some studies showed intact non-declarative memory functions such as implicit sequence learning^10–14^ and priming^5^ in older individuals and those with AD^15,16^, others reported age- or disease-related deficits^6,17–20^. The latter can lead to difficulties in adapting to new tasks, acquiring new skills, and retaining previously acquired information.

To investigate implicit learning more specifically, prior studies used the Alternating Serial Reaction Time (ASRT) task to separate two fundamental underlying aspects, known as general visuomotor learning and probabilistic sequence-specific learning^18^. The former describes the ability to respond faster to a sequence of stimuli through practice, where the structure of the sequence is irrelevant. The latter expounds upon the unconscious acquisition of knowledge regarding the intrinsic sequence structure, leading to accelerated responsiveness to elements that possess higher probability of occurrence. The distinct impacts of advancing age on both aspects were illustrated in two previous lifespan studies. Janacsek et al.^19^ demonstrated a progressive augmentation of sequence-specific learning during early childhood, culminating in peak performance around the age of 12, followed by a subsequent decline with increasing age. In their study, Juhasz et al.^21^ not only successfully replicated the previous sequence-specific learning findings but also revealed a U-shaped developmental trajectory in visuomotor learning. Interestingly, both children and older adults displayed an enhanced learning effect in this aspect, which contrasts with the developmental curve observed in sequence-specific learning. The investigation of how these two facets of implicit learning unfold in the context of cognitive decline was elucidated in a study involving patients diagnosed with MCI. Nemeth et al.^20^ reported impaired probabilistic sequence-specific learning for MCI patients compared to healthy controls, but only in the reactivation phase of the learning process. Furthermore, while controls showed a significant offline (between practice sessions) visuomotor learning after 24 h, MCI patients did not. These studies, through the application of the ASRT task, showed the distinct effects of aging and cognitive decline on different facets of implicit learning.

To address the challenges posed by age- or disease-related decline in memory functions, researchers have been exploring innovative methods for cognitive enhancement. One promising approach is the use of Non-invasive Brain Stimulation (NiBS) techniques, such as transcranial Alternating Current Stimulation (tACS). By modulating neural oscillations, tACS has shown the potential in enhancing cognitive functions across various cognitive domains^22–24^. In the context of memory, most tACS studies aimed to improve either Working Memory (WM)^25–33^ or declarative memory^34–36^. Although study designs vary widely with respect to the underlying parameters used in the application of tACS (intensity, duration, frequency, number of stimulations, montage, target structure), making results difficult to compare, especially the studies in which older participants who already exhibited more or less severe cognitive decline were treated, show a potentially positive effect of tACS on WM, or declarative memory^27,35,36^.

To date, only few studies have investigated a possible effect of tACS on implicit learning^37–42^ targeting either general visuomotor learning^37,39,40^, sequence-specific learning^41,42^ or both aspects^38^. Authors suggest a possible modulation of both learning aspects through tACS, with the effect either facilitating or inhibiting learning depending on the frequency used in the tACS paradigm, the brain structure targeted, and the participants’ age. For instance, stimulation of the primary motor cortex (M1) with alpha (10 Hz) tACS resulted in enhanced visuomotor learning^37^. Another study targeting M1 demonstrated improved sequence-specific learning following alpha (10 Hz) or beta (20 Hz) tACS, while gamma (35 Hz) tACS or sham tACS did not entail any modulation^41^. Furthermore, Fresnoza et al.^38^ documented an age-dependent effect of tACS on implicit learning. Their findings revealed enhanced consolidation of visuomotor and sequence-specific skills among older adults, while the intervention tended to impair performance in young participants. On the other hand, Zavecz et al.^42^ failed to influence probabilistic sequence-specific learning through fronto-parietal midline theta (6 Hz) tACS in healthy young adults. However, except for Zavecz et al.^42^ which is the only study to date that employed the ASRT task to explore a possible modulation of implicit learning by tACS, the results of the remaining studies rely on the Serial Reaction Time Task (SRTT). Unlike the ASRT task, which involves a sequence comprising alternating predetermined and random elements, referred to as probabilistic second-order dependency, the SRTT entails a repetitive sequence consisting solely of predetermined elements^18,43^. While the former (referring to the non-adjacent probabilities) ensures that participants remain entirely unaware of the sequence structure, making it completely implicit, it is important not to discount the possibility of explicit knowledge accumulation in the SRTT^44^.

In this study, we applied bilateral tACS over the Dorsolateral Prefrontal Cortex (DLPFC) using the novel peak-coupled theta—gamma cross-frequency waveform, which was prior shown to be more effective than single theta tACS or trough-coupled theta—gamma tACS in boosting cognitive function and motor skill learning^26,45^. We chose to target the DLPFC due to its involvement in implicit sequence learning^46–48^ while being not only penetrable by tACS, but also representing the most promising target for improving cognition in older adults^49^. During the course of stimulation, participants engaged in a Computerized Cognitive Training (CCT) paradigm aimed at regulating the neurophysiological state (keeping the state-of-the-brain comparable among participants), which is another important factor in the design of tACS experiments, as it may influence the efficacy of tACS through ongoing activity in the targeted brain area^50–53^. We hypothesized that promoting synchronization between theta and gamma oscillations in the DLPFC through repeated tACS may enhance non-declarative memory functions in older participants.

## Materials and methods

### Participants

According to our inclusion criteria, all participants were neurologically and psychiatrically healthy, did not suffer from severe internal diseases, nor had they undergone chemotherapy within the last 12 months. According to the safety aspects of using NiBS techniques, it was ensured that no one had ever had an epileptic seizure or stroke and that no pacemaker was implanted nor metal implants in the brain, although these are not absolute contraindications^54^. All participants were German native speakers without dependence problems on alcohol, medication or other drugs. Participants who were under active treatment with neuroleptics, benzodiazepines or antiepileptics at the time of the study were excluded beforehand. All participants gave written informed consent.

In total, 45 participants were recruited for this study in Göttingen, Germany. Of these, ten participants revised their participation due to various reasons (see Supplementary Materials for the dropout details, S1.1), leaving a final sample of 35 older adults (19 female, age range 55–82 years, mean age 69.5 ± 6.8 years), who were randomly divided into two intervention groups, either active tACS (17 participants) or sham tACS (18 participants). Age, gender, education and baseline cognitive state (see section “*Montreal Cognitive Assessment*”) were matched between groups (see Table 1 for the baseline characteristics).

**Table 1:** Baseline characteristics of the intervention groups.

	Sham tACS (n = 18)	Active tACS (n = 17)	p value
Age, years	69.9 (6.9)	69 (6.8)	0.70
Gender (F/M)	9/9	10/7	0.85
Education, years	12.9 (3.3)	14 (6.1)	0.51
MoCA, baseline	24.7 (3.1)	25.2 (2.8)	0.57

Data represents the mean values and standard deviations (in parentheses) for age (in years), years of education, and Montreal Cognitive Assessment (MoCA) scores. For gender (F, female; M, male), case numbers are given. The column labeled "p value" presents the observed distinctions between the intervention groups at baseline. To assess the variables of age, education, and MoCA, two-sample two-sided t-tests were employed. The comparison of gender distribution was conducted using the chi-squared (χ^2^) test. A p value ≤ 0.05 indicates statistical significance.

### Study design

**Figure 1:**
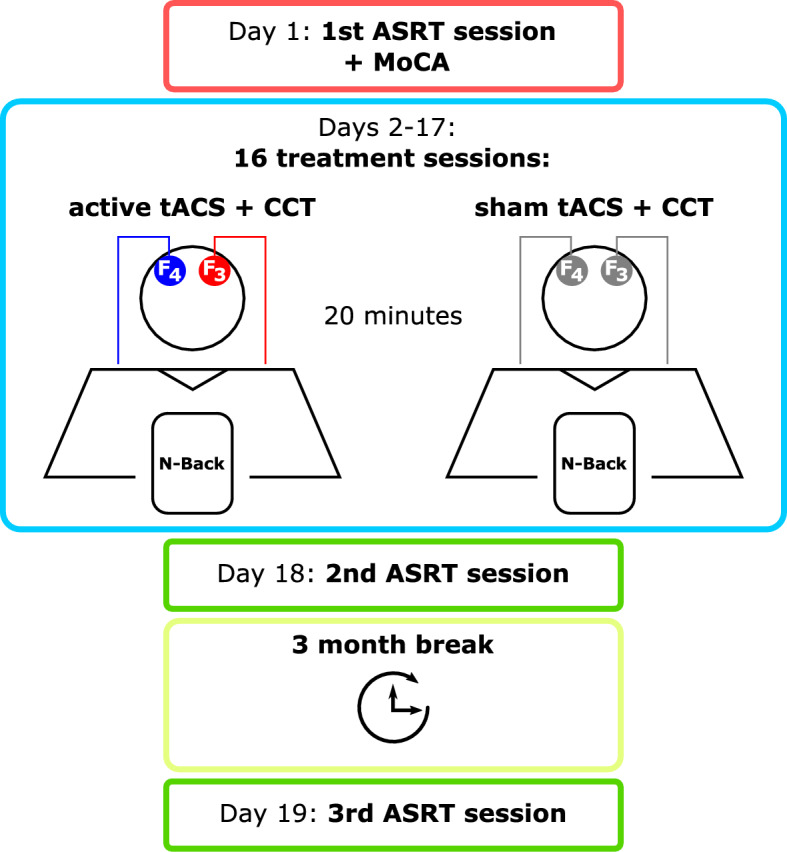
Study design. In this triple-blinded, placebo-controlled, parallel-group research study, participants came to the clinic for 19 appointments. The first appointment included history taking, implementation of the Montreal Cognitive Assessment (MoCA), and an initial administration of the Alternating Serial Reaction Time (ASRT) task (outlined in red). At each of the subsequent 16 appointments, participants received 20 min of active or sham tACS treatment bilaterally targeting the dorsolateral prefrontal cortex (F3/F4, according to the international 10–20 system) while completing Computerized Cognitive Training (CCT) based on the n-back paradigm on a tablet (outlined in blue). After completion of treatment, two further appointments were held to examine direct treatment effects and long-term effects (outlined in green), separated by a 3-month break (outlined in yellow).

We conducted a randomized (1:1), triple-blinded, placebo-controlled, parallel-group research study. Before starting the treatment, each participant completed an initial cognitive assessment, which included the ASRT task and the Montreal Cognitive Assessment (MoCA). Subsequently, each participant attended 16 treatment sessions over a period of 6 weeks, with the aim to induce longer lasting effects. In the treatment sessions, participants were stimulated with active or sham tACS for 20 min while concurrently performing tablet-based CCT. The second and third ASRT session took place directly after and 3 months after completion of treatment, respectively, to measure both direct and long-term effects (Fig. 1). The study was approved by the Ethics Committee of the medical faculty of the Georg-August-University, Göttingen, Germany (7/7/2020) and the interventions were applied according to the Declaration of Helsinki.

### Cognitive assessment

#### Alternating serial reaction time task

**Figure 2:**
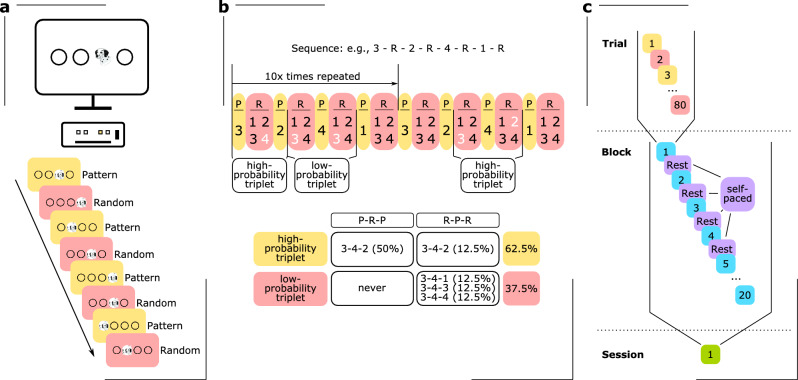
The Alternating Serial Reaction Time (ASRT) task. (**a**) Participants were placed in front of a screen and provided with a keyboard containing only four keys. Each key corresponded to one of four circles displayed horizontally on the screen. When a stimulus (a dog's head) appeared in any of the circles, the participant's task was to press the corresponding key as fast as possible. Once the correct key was pressed, a new stimulus appeared. The position of the stimulus alternated between predetermined *pattern* (P, yellow background) and *random* (R, red background) elements. (**b**) The predetermined pattern elements were part of an eight-element sequence, in which they alternated with random elements (e.g. 3-R-2-R-4-R-1R). The numbers represented the corresponding position on the screen in ascending order from left to right. Every trial was characterized as the third element of the underlying triplet (three consecutive trials). Due to the probabilistic sequence structure, this results in so-called high-probability triplets, which occur with a probability of 62.5%, and low-probability triplets, which occur with a probability of 37.5%^75^. (b) One ASRT session (green background) consisted of 20 blocks (blue background), each containing 80 trials (yellow and red background), which was ten times the repetition of the eight-element sequence. Between blocks, participants could take self-paced breaks (purple background).

To measure different aspects of implicit learning, we used the ASRT task^18^, which was programmed in JavaScript using the jsPsych library v.6.1.0^55,56^. In each trial of the ASRT task, a stimulus (a drawing of a dog’s head) appeared on the screen in one of four horizontal positions and remained there until the participant provided a response. The participants were instructed to press the key corresponding to the location of the target stimulus on the screen as fast as possible using only their left and right middle and index fingers. To avoid careless errors, all unnecessary keys were removed from the keyboard, leaving only four keys that represent the four positions on the screen (S, F, J and L) (Fig. 2a).

The stimuli adhered to an eight-element sequence characterized by probabilistic patterns, alternating between pattern ("P") and random ("R") elements (e.g., 3-R-2-R-4-R-1-R, where the numbers signify prearranged positions from left to right). Due to the probabilistic sequence structure, certain triplets (i.e., three consecutive elements) have a higher probability to occur than others. The so-called high-probability triplets (hpt) have a probability of 62.5% and can either be structured as P-R-P or as R-P-R, while the so-called low-probability triplets (lpt) have a probability of 37.5% and can only be structured as R-P-R. This eight-element sequence was repeated ten times in each block (Fig. 2b). Participants were not provided with prior knowledge regarding the specific structure of the sequence or the occurrence of repetitions.

All participants underwent two blocks of random trials at the beginning of the experiment to familiarize themselves with the task, which were later excluded from the analysis. After the practice, each session of the ASRT task consisted of 20 blocks, each containing 80 trials. Between blocks, participants could take self-paced breaks (Fig. 2c).

After the last ASRT session, participants performed a short questionnaire confirming the unawareness about the sequence repetition. Even though some participants answered in the affirmative to the questions "Have you noticed anything special regarding the task?", and "Have you noticed some regularity in the sequence of stimuli?", the sequences given did not correspond to reality in any case, therefore, we can confirm that the pattern was not recognized by any of the participants.

#### Montreal cognitive assessment

The MoCA task is a fast screening tool for cognitive disorders such as MCI or AD targeting different cognitive domains such as visuospatial abilities, executive functions, short-term memory, WM, attention, language and orientation to time and place^57^. Since the clinical routine for MCI diagnosis includes physical and imaging examinations in addition to neurophysiological testing^58^, the participants in this study were not classified as either healthy or mild cognitively impaired based on their individual MoCA score. Rather, the MoCA test was used to correlate the results with the respective cognitive level.

### Transcranial alternating current stimulation

TACS is a safe method for non-invasive stimulation of target areas in the brain using low intensity (max. 4 mA peak-to-peak) alternating electric currents^54^. Depending on the specific montage, at least two electrodes are required, which are attached to the scalp and fixed with elastic rubber bands. The current flowing between these two electrodes induces an electric field in the brain areas located between the electrodes. In our setting, we used a bilateral montage placing two conductive rubber electrodes (⌀ 2 cm) at F3 and F4 according to the international 10–20 system, targeting the DLPFC. This electrode placement represents the most widely used tACS montage for improving cognitive functions^59^. To maximize the conductivity and minimize the impedance of the electrode–skin junction, the skin under the electrodes was cleaned in advance by skin disinfectant and the electrodes were coated with a small amount (approximately 1 mm thickness) of conductive cream (ac cream, Spes Medica S.r.l., Genova, Italy) such that the electrodes were completely covered by the cream, but the cream did not protrude excessively after fixing. Using the CE-certified neuroConn DC-STIMULATOR MOBILE (neurocare group AG, Ilmenau, Germany), a 2 mA (peak-to-peak) alternating current was delivered to the brain for 20 min (15 s fade in and 15 s fade out), while the impedance was kept below 15 kΩ. For the tACS frequency, we used a theta—gamma cross-frequency coupling paradigm based on a slow theta wave (6 Hz), superimposed by high gamma bursts (80 Hz) only on the peaks of the theta wave, which was previously shown to optimally boost working memory improvement compared to single theta tACS or theta—gamma tACS with gamma bursts coincided with the troughs of the theta wave^26^. This paradigm relies on phase—amplitude coupling, wherein the phase of the theta wave modulates the amplitude of the superimposed gamma oscillation. In our tACS setup, the gamma bursts reached a maximum intensity of 0.9 mA peak-to-peak at the peaks of the underlying theta wave. Specifically, six gamma cycles were superimposed onto one theta peak. In the sham tACS protocol, the current was delivered only in the beginning and in the end of the stimulation period, both for 90 s each (15 s fade in, 60 s active stimulation with 2 mA peak-to-peak, 15 s fade out). Between these blocks, a continuous, sinusoidal 85 Hz current with a low intensity of 50 μA peak-to-peak was applied to ensure that there were no sham-induced effects on cortical excitability^60^. In contrast to the most commonly used sham protocol for transcranial Electrical Stimulation (tES), in which the active stimulation lasts for 30 s only and only in the beginning of the stimulation period^61^, we doubled the active stimulation time and added an additional stimulation block at the end of the treatment duration to overcome the problem of the previously described insufficient blinding when the stimulation is repeated and administered for 20 min^62^. The experimenters who applied the stimulation and the data analyst were also blinded to the type of stimulation, thus ensuring triple-blinding. Coding of the stimulators was performed by a third person who was not involved in data collection or analysis. In total, each participant was stimulated with either active or sham tACS for 320 min, separated into 16 sessions over a period of 6 weeks (2–3 sessions/week). Due to the high number of sessions, we opted for group therapies with up to four participants per session.

### Computerized cognitive training

Synchronous to tACS, participants underwent an intensive CCT paradigm to regulate their neurophysiological state by forcing their attention to the performance of two n-back games. During the first and the last third of the stimulation period, participants were instructed to play a 2-back game (*Memory Match*), whereas between these blocks a 1-back game was performed (*Speed Match*). Both games were provided as part of a research collaboration with Lumos Labs, Inc., incorporated into their brain jogging app Lumosity™, and were run on tablets (Samsung Galaxy Tab A). Both are virtual card games, where the user has to decide on the basis of “Yes” and “No” whether the current stimulus (a certain card) matches the stimulus n-steps before. In total, there are five different cards in each of the two games, which can be distinguished by shape or color: green flower, yellow triangle, red square, purple diamond, blue circle. The n-back task is generally considered to be the most widely used WM training paradigm^63^.

### Statistical analyses

Before analysis, data originating from the initial two practice blocks, consisting of 160 trials per participant and assessment, were subjected to exclusion, along with the first two stimuli from each block (40 trials per participant and assessment). Additionally, trills (e.g. 1-2-1, 4-1-4, where numbers indicate horizontal positions from 1 to 4) and repetitions (e.g. 1-1-1, 3-3-3) were excluded from the raw ASRT data set^17^. Through this process, 22% of the original trials were eliminated. Then, reaction times and accuracies were calculated separately for each participant, each unit of five blocks (referred to below as epoch), each session and for both high- and low-probability triplets, based on the median of correct responses and the mean of all responses, respectively. Given the consistent high accuracy exhibited by the participants in both intervention groups across all three test time points (average exceeding 95%), leading to a ceiling effect, the subsequent evaluation disregarded the analysis of accuracy, as commonly observed in previous studies examining the ASRT task^18,20,64^. All statistical analyses described below have been performed in R^65^ using the R Studio IDE^66^.

To investigate the effects of treatment in the context of aging, we fitted different Linear Mixed Models (LMMs) using the lme4 package^67^ to predict RT with the following variables: session (1–3), epoch (1–4), trial type (hpt vs. lpt), group (active tACS vs. sham tACS), MoCA (1–30) and age (55–85). Using the ASRT task, we were able to differentiate between two types of learning: probabilistic sequence-specific learning and visuomotor learning. Moreover, we separated effects that occur during practice sessions (online) and effects that occur between practice sessions (offline). In our models, *online probabilistic sequence-specific learning* was indicated by a significant effect of trial type on RT, such that RT is lower for hpt than for lpt. Enhancements observed across sessions (significant interaction between trial type and session) were termed *offline probabilistic sequence-specific learning*. Decreasing RT throughout the execution of the ASRT task (significant effect of epoch) referred to *online visuomotor learning,* whereas faster RT between practice sessions was termed *offline visuomotor learning.*

By using the *report* package to report our LMM results, including the model's explanatory power (conditional R^2^, marginal R^2^), slope (beta), 95% confidence interval (CI), t-value, p value, standardized beta (std. beta) and standardized CI (std. CI), we followed best practice guidelines aimed at standardization, comparability and reproducibility in reporting results^68^. Standardized parameters were obtained by fitting the model on a standardized version of the dataset. 95% CIs and p values were computed using a Wald t-distribution approximation. Reproducible LMM summary tables were created using the *gtsummary* package^69^ and later supplemented with additional information. All assumptions for LMMs, such as linearity, normality, homoscedasticity, collinearity and influential data points, were satisfied for the models used in this work. To examine, which factors (or interaction of factors) were significantly affecting the quality of the model, we applied the Likelihood ratio test (using the *anova* function in R) comparing the likelihoods, which are represented by the Akaike Information Criteria (AIC) in LMMs, of two or more models with each other. Therefore, a significant p value indicated a significant effect of the particular term added compared to the model without that term and according to the minimum AIC procedure, the model with the lowest AIC was considered the best model with the highest information gain^70^. Figures were created using the *ggplot2*, *ggpubr* and *sjplot* packages^71–73^.

## Results

### Can we enhance implicit learning in older adults with repeated theta—gamma tACS?

To explore the treatment effects of tACS on both aspects of implicit learning—sequence-specific learning and visuomotor learning—we fitted different LMMs testing the importance of session (1–3), epoch (1–4), trial type (hpt vs. lpt) and group (active tACS vs. sham tACS) (and interactions) to predict RT. All models included a random intercept for participant and a by-participant random slope for the effect of epoch (formula: ~ 1 + epoch | participant). Subsequently, LMMs were compared utilizing the minimum AIC procedure. Our final best model (formula: RT ~ group * session + trial type + epoch * session) (Table 2) with the lowest AIC (8119) revealed *online sequence-specific learning* such that participants were faster on hpt than on lpt*,* which was indicated by a statistically significant and positive effect of trial type [lpt] (beta = 7.33, 95% CI [3.75, 10.90], t(826) = 4.02, p < 0.001; Std. beta = 0.09, Std. 95% CI [0.05, 0.13]). Adding a trial type × session or trial type × session × group interaction term increased the AIC of the model (8123.2; 8127.4) while being non-significant (p = 0.971; p = 0.877), respectively. Thus, neither active nor sham tACS could modulate sequence-specific learning.

**Table 2:** Results of the linear mixed model, predicting median RTs to investigate tACS treatment effects.

Predictor	N	Beta	95% CI	Std. Beta	Std. 95% CI	p value
Group						0.4
Sham tACS	432	–	–	–	–	
Active tACS	408	19	− 27, 64	0.23	− 0.30, 0.76	
Session						
1	280	–	–	–	–	
2	280	− 44	− 52, − 35	− 0.33	− 0.41, − 0.26	** < 0.001**
3	280	− 57	− 65, − 49	− 0.53	− 0.60, − 0.45	** < 0.001**
Trial type						** < 0.001**
hpt	420	–	–	–	–	
lpt	420	7.3	3.8, 11	0.09	0.05, 0.13	
Epoch	840	− 13	− 17, − 8.9	− 0.17	− 0.22, − 0.12	** < 0.001**
Group * session	840					
Active tACS * 2	136	− 21	− 30, − 12	− 0.25	− 0.36, − 0.15	** < 0.001**
Active tACS * 3	136	− 21	− 29, − 12	− 0.25	− 0.35, − 0.14	** < 0.001**

The model's total explanatory power was substantial (conditional R^2^ = 0.90) and the part related to the fixed effects alone (marginal R^2^) was 0.09. The Akaike Information Criterion (AIC) of the model was estimated by the maximum likelihood method and was 8119. Standardized parameters were obtained by fitting the model on a standardized version of the dataset. 95% CIs and p values were computed using a Wald t-distribution approximation. A p value ≤ 0.05 indicates statistical significance (highlighted in bold).

*CI* confidence interval.

Conditional R^2^ = 0.90.

Marginal R^2^ = 0.09.

AIC = 8119 (estimated using *Maximum Likelihood*).

**Figure 3:**
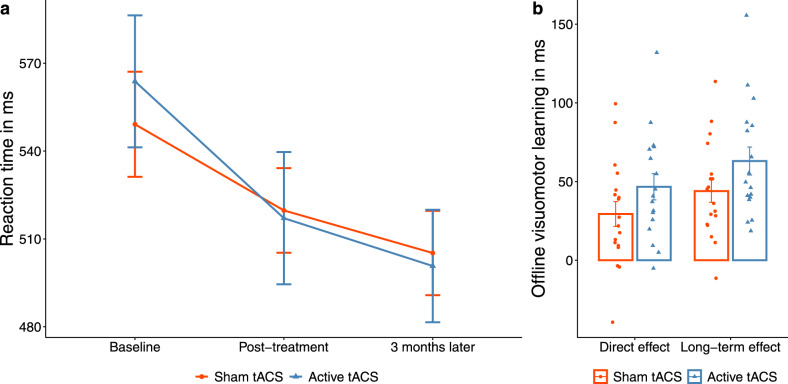
Active theta—gamma transcranial Alternating Current Stimulation (tACS) boosts offline visuomotor skill learning. (**a**) Participants from the active tACS group (blue line, n = 17) exhibited greater decrease in reaction time from baseline to the post-treatment and to the follow-up (3 months later) assessments, compared to participants from the sham tACS group (orange line, n = 18). (**b**) This resulted in greater direct (measured as the RT difference between baseline and post-treatment) as well as long-term (measured as the RT difference between baseline and follow-up) offline visuomotor learning in the active tACS group (blue bars), compared to the sham tACS group (orange bars). The error bars represent the standard error of the mean (SEM).

The model also revealed *online* and *offline visuomotor learning* such that RT decreased throughout the task, which was shown by a statistically significant and negative effect of epoch (beta = − 12.70, 95% CI [− 16.45, − 8.95], t(826) = − 6.64, p < 0.001; Std. beta = − 0.17, 95% CI [− 0.22, − 0.12]) and across sessions, which was indicated by a statistically significant and negative effect of session [2] (beta = − 43.55, 95% CI [− 52.02, − 35.08], t(826) = − 10.09, p < 0.001; Std. beta = − 0.33, Std. 95% CI [− 0.41, − 0.26]) and session [3] (beta = − 57.03, 95% CI [− 65.50, − 48.56], t(826) =  − 13.21, p < 0.001; Std. beta = − 0.53, Std. 95% CI [− 0.60, − 0.45]). Moreover, while treatment groups exhibited no significant RT differences in general, which was indicated by a non-significant and positive effect of group [active tACS] (beta = 18.78, Std. 95% CI [− 25.09, 62.65], t(826) = 0.84, p = 0.401; Std. beta = 0.23, 95% CI [− 0.30, 0.76]), participants in the active tACS group revealed a significantly greater offline visuomotor learning across sessions compared to those in the sham tACS group, which was shown by a statistically significant and negative interaction effect of group [active tACS] × session [2] (beta = − 21.01, 95% CI [− 29.77, − 12.26], t(826) =  − 4.71, p < 0.001; Std. beta = − 0.25, Std. 95% CI [− 0.36, − 0.15]) and a statistically significant and negative interaction effect of group [active tACS] × session [3] (beta = − 20.57, 95% CI [− 29.33, − 11.81], t(826) =  − 4.61, p < 0.001; Std. beta = − 0.25, Std. 95% CI [− 0.35, − 0.14]) (Fig. 3). Our results demonstrated that repeated theta—gamma tACS over the DLPFC improved visuomotor learning but did not influence probabilistic sequence-specific learning in older adults. Full LMM results (Table S2.1) and further findings (S.2.1) can be found in the Supplementary Materials.

### Does the effectiveness of tACS treatment vary with age or cognition?

**Figure 4:**
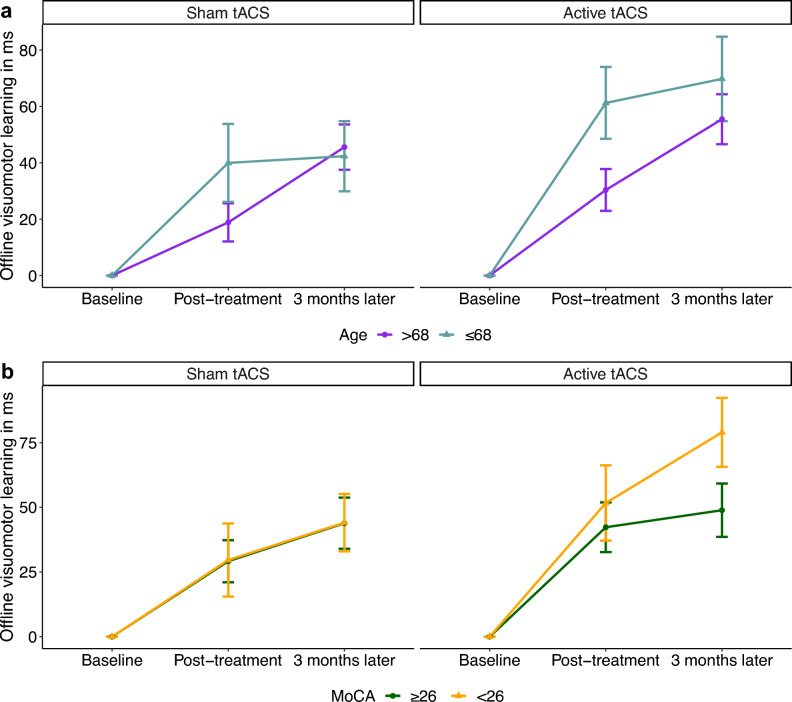
Active tACS treatment is more effective in younger and cognitively weaker participants. (**a**) In the active tACS group (upper right plot), older adults of younger age (≤ 68 years, light blue line, n = 18) revealed higher offline visuomotor learning than those of older age (> 68 years, purple line, n = 17) 3 months after treatment completion, whereas in the sham tACS group (upper left plot), both age groups showed the same level of offline visuomotor learning 3 months after treatment. (**b**) In the active tACS group (lower right plot), participants with MoCA (i.e. Montreal Cognitive Assessment) scores in the range of Mild Cognitive Impairment (MCI) (< 26, orange line, n = 17) revealed higher offline visuomotor learning than participants with MoCA scores in the healthy range (≥ 26, dark green line, n = 18) 3 months after treatment completion, whereas in the sham tACS group (lower left plot), both cognitively healthy and cognitively impaired participants exhibited the same level of offline visuomotor learning 3 months after treatment. The error bars represent the standard error of the mean (SEM).

To examine correlations between treatment effects and various aspects of aging, we included MoCA (1–30) and age (55–85) as additional variables into our model fitting process. Our final best model (formula: RT ~ group * session * MoCA + trial type + group * session * age + epoch * session * MoCA) (Table 3) with the lowest AIC (8051) revealed higher offline visuomotor learning for younger participants directly after completion of treatment, which was indicated by a statistically significant and positive interaction effect of session [2] × age (beta = 1.40, 95% CI [0.52, 2.28], t(811) = 3.13, p = 0.002; Std. beta = 0.11, 95% CI [0.04, 0.18]), but not in the long term, which was shown by a statistically non-significant and negative interaction effect of session [3] × age (beta = − 0.58, 95% CI [− 1.46, 0.30], t(811) =  − 1.28, p = 0.199; Std. beta = − 0.05, 95% CI [− 0.12, 0.02]). In addition, a statistically significant and positive three-way interaction of (group [Active tACS] × session [3]) × age (beta = 1.94, 95% CI [0.55, 3.32], t(811) = 2.74, p = 0.006; Std. beta = 0.16, 95% CI [0.04, 0.27]) revealed higher offline visuomotor learning 3 months after treatment in younger participants from the active tACS group compared to those from the sham tACS group (Fig. 4a).

**Table 3:** Results of the linear mixed model, predicting median RTs to investigate correlations between tACS treatment efficacy, age and baseline cognition.

Predictor	N	Beta	95% CI	Std. Beta	Std. 95 CI	p value
MoCA	840	− 15	− 25, − 6.5	− 0.38	− 0.68, − 0.08	**< 0.001**
Age	840	2.6	− 1.22, 6.4	0.21	− 0.1, 0.51	0.183
Group * MoCA	840					0.6
Active tACS * MoCA	408	− 4.2	− 19, 11	− 0.15	− 0.64, 0.35	
Session * MoCA	840					
2 * MoCA	280	6.8	4.0, 9.6	0.09	0.02, 0.16	** < 0.001**
3 * MoCA	280	4.6	1.9, 7.4	0.05	− 0.02, 0.12	**0.001**
Session * age	840					
2 * age	280	1.1	0.52, 2.3	0.11	0.04, 0.18	**0.002**
3 * age	280	− 0.58	− 1.5, 0.3	− 0.05	− 0.12, 0.02	0.199
Group * age	840					0.5
Active tACS * age	408	2.0	− 4.2, 8.2	0.16	− 0.32, 0.65	
Group * session * MoCA	840					
Active tACS * 2 * MoCA	136	− 2.3	− 5.5, 0.99	− 0.08	− 0.19, 0.03	0.172
Active tACS * 3 * MoCA	136	3.3	− 0.01, 6.5	0.11	0.0, 0.23	0.051
Group * session * age	840			0.011		
Active tACS * 2 * age	136	0.23	− 1.2, 1.6	0.02	− 0.09, 0.13	0.743
Active tACS * 3 * age	136	1.9	0.55, 3.3	0.16	0.04, 0.27	**0.006**

The model's total explanatory power was substantial (conditional R^2^ = 0.91) and the part related to the fixed effects alone (marginal R^2^) was 0.47. The Akaike Information Criterion (AIC) of the model was estimated by the maximum likelihood method and was 8051. Standardized parameters were obtained by fitting the model on a standardized version of the dataset. 95% CIs and p values were computed using a Wald t-distribution approximation. A p value ≤ 0.05 indicates statistical significance (highlighted in bold).

*CI* confidence interval.

Conditional R^2^ = 0.91.

Marginal R^2^ = 0.47.

AIC = 8051 (estimated using *Maximum Likelihood*).

The model also showed that participants with lower MoCA scores at baseline had higher offline visuomotor learning immediately after completing treatment and 3 months later, which was indicated by a statistically significant and positive interaction effect of session [2] × MoCA (beta = 6.79, 95% CI [4.03, 9.56], t(811) = 4.82, p < 0.001; Std. beta = 0.09, 95% CI [0.02, 0.16]) and session [3] × MoCA (beta = 4.64, 95% CI [1.87, 7.40], t(811) = 3.29, p = 0.001; Std. beta = 0.05, 95% CI [− 0.02, 0.12]). In addition, a nearly significant and positive three-way interaction of (group [Active tACS] × session [3]) × MoCA (beta = 3.26, 95% CI [− 0.01, 6.53], t(811) = 1.95, p = 0.051; Std. beta = 0.11, 95% CI [− 4.65e−04, 0.23]) indicated higher offline visuomotor learning 3 months after treatment in participants with lower MoCA from the active tACS group compared to those from the sham tACS group (Fig. 4b). Our results showed that active multi-session tACS treatment led to higher long-term offline visuomotor learning in younger and cognitively weaker participants. Full LMM results (Table S2.2) and further findings (S.2.2) can be found in the Supplementary Materials.

### Only mild adverse effects and successful blinding

After each tACS session, participants were asked about Adverse Effects (AEs). The number of reported AEs were similar in both active (tingling = 193, skin irritations = 2, headache = 5, tiredness = 2) and sham (tingling = 225, skin irritations = 8, headache = 2, tiredness = 4) tACS groups. This was confirmed by the unpaired two-sided t-test (tingling: p = 0.35; skin irritations: p = 0.28; headache: p = 0.27; tiredness: p = 0.5). Phosphenes only occurred in the sham group (phosphenes = 7) (see Supplementary Materials for an overview of AEs, S1.2). Thus, only mild AEs but no moderate or serious AEs were reported and no differences were observed between intervention groups.

Moreover, after completion of the stimulation series, participants were asked to guess which type of stimulation they received. Possible answers were: “Active”, “Sham” or “I don’t know”. In total, 18 out of 35 participants guessed right (51%). Successful blinding was confirmed by Pearson’s Chi-squared test revealing independence of the guesses from the real condition (χ^2^ = 1.05, p = 0.59), followed by the Fisher’s Exact Test (p = 0.72) due to the small number (< 5) of” “I don’t know” guesses in both groups. Therefore, participants were not able to distinguish between active and sham tACS.

## Discussion

In this study, we aimed to enhance non-declarative memory functions in older adults through repeated peak-coupled theta—gamma tACS over the DLPFC. To examine the effects of tACS treatment on different aspects of implicit learning, we applied the ASRT task, which allows us to distinguish between general visuomotor learning and probabilistic sequence-specific learning. The results of our randomized, triple-blinded research study provided evidence for the successful modulation of visuomotor learning, indicating higher learning rates in the active tACS group immediately after completing 16 treatment sessions as well as 3 months later, compared to the sham tACS group. We did not observe any effect of stimulation on probabilistic sequence-specific performance. In addition, active tACS treatment was more effective in older adults of younger age and those with poorer cognition at baseline, leading to greater offline consolidation of visuomotor learning in the long-term.

Although studies have already demonstrated the potential of peak-coupled theta—gamma tACS to improve working memory^26^ or motor skill acquisition^45^, we are the first to test this stimulation paradigm to improve non-declarative memory functions. Our results corroborate the findings of Akkad et al.^45^ by revealing enhanced visuomotor learning subsequent to active theta—gamma tACS as opposed to sham tACS, providing empirical support for the involvement of theta—gamma coupling in the process of motor learning. On the other hand, the non-modulation of probabilistic sequence-specific learning indicates a selective involvement of theta—gamma coupling in distinct cerebral functions, thereby necessitating separate consideration of sequence-specific learning and visuomotor learning. Prior investigations reported no behavioral effects in terms of verbal declarative memory^34^ and cognitive control^74^ subsequent to peak-coupled theta—gamma tACS. Interestingly, trough-coupled theta—gamma tACS resulted in negative behavioral effects in both studies. Hence, mixed results emerge about the effects of theta—gamma tACS, highlighting the need for further research to elucidate the complex interplay between theta—gamma coupling, the phase-specificity and diverse cognitive tasks.

To date, only one previous study similarly aimed to modulate implicit learning based on the ASRT task using tACS. Zavecz et al.^42^ reported no modulation of probabilistic sequence-specific learning by fronto-parietal midline theta tACS in young adults but did not examine effects on visuomotor learning. Compared to the prior work, we adjusted some of the tACS parameters by doubling the intensity (2 mA peak-to-peak), applying theta—gamma cross-frequency coupling, and targeting the DLPFC. Another difference between the two studies is that we measured offline effects of repeated stimulation, whereas the results of Zavecz et al. were based on single session tACS during the execution of the ASRT task. In addition, we applied a CCT paradigm simultaneously with stimulation to control the participants' brain state. Notably, we focused on older adults, thereby expecting higher learning potential and less ceiling effects, whereas Zavecz et al.^42^ recruited healthy young adults. While diverging significantly in various aspects that complicate direct comparison, both studies failed to affect probabilistic sequence-specific learning through tACS. However, the multitude of potential actuators listed above illustrates possible reasons contributing to the absence of anticipated modulation effects that will be elaborated upon in the subsequent paragraphs.

So far, targeting the DLPFC with neuromodulation methods to affect implicit sequence-specific learning has yielded conflicting results. A previous study by Janacsek et al.^75^, in which anodal transcranial Direct Current Stimulation (tDCS) was applied over the DLPFC during the ASRT task, reported a greater learning effect with anodal stimulation of the right DLPFC compared to sham tDCS, whereas stimulation of the left DLPFC showed no effect on sequence-specific learning. The authors concluded an important role of the right fronto-striatal network in statistical learning. Another study reported improved sequence-specific learning after disruption of the DLPFC with 1 Hz rTMS that is supposed to have inhibitory effect on neuronal activity^76^. In contrast, studies by Savic et al.^77–79^ found no effects of anodal or cathodal tDCS or focal high-definition tDCS over the left or right DLPFC on implicit sequence learning and consolidation. However, in all three studies, Savic et al. applied a task sequence learning paradigm to study implicit sequence learning, which, unlike the ASRT task used in the studies of Janacsek et al.^75^ and Ambrus et al.^76^, does not have a probabilistic sequence structure. Previous imaging studies support the findings of Janacsek et al.^75^ and Ambrus et al.^76^ by demonstrating the involvement of the DLPFC in implicit sequence learning^46,47^. However, further aspects and their interplay, such as the targeted hemisphere, the applied NiBS method and the specific sequence learning approach seem to play a role in this neuromodulation process.

Among all studies that have aimed to modulate probabilistic sequence-specific learning using NiBS methods, we are, to our knowledge, the first ones to have investigated offline (between practice periods) rather than online (during practice) effects. Prior investigations exploring the impact of an offline interval (12 h, 24 h, 1-week) on the consolidation of probabilistic sequence-specific knowledge and visuomotor learning consistently demonstrated the lack of “silent” enhancement in the former, contrasting with its evident occurrence in the latter^64,80–82^. In our study design, the first tACS session typically commenced after a minimum of 24 h following the completion of the baseline ASRT task. Hence, we posit that the previously acquired sequence-specific knowledge had already undergone "decay" prior to the onset of stimulation. On the other hand, our results add to the body of knowledge by demonstrating successful offline consolidation of visuomotor learning. Notably, the observed augmentation in visuomotor learning subsequent to active theta—gamma tACS treatment provides suggestive evidence for its potential to enhance offline consolidation of this specific implicit learning component. However, our findings diverge from the results reported by Nemeth and Janacsek^81^ as we identified significant offline visuomotor learning in older adults also in the sham tACS group at both the 6-week and 3-month time points. In contrast, Nemeth and Janacsek observed offline visuomotor learning in older adults only after a 12-h interval, without significant findings after 24 h or 1 week. A possible explanation is offered by the CCT, which took place equally in both intervention groups in our study, i.e. also in the sham tACS group, and could also contribute to the consolidation of visuomotor learning. Recent research already demonstrated the feasibility of the n-back task to enhance motor learning^83^, which was mainly justified by an attention-enhancing effect that can be observed after repeated n-back training^63^. Additional research utilizing a control group receiving no treatment could significantly enhance the strength of these conclusions.

Previous studies demonstrated that age^38,84–87^ and the cognitive level^88,89^ may influence the effectiveness of tES interventions. We can corroborate the aforementioned studies by demonstrating that younger participants and those afflicted with pre-existing cognitive decline exhibit a more pronounced tACS treatment effect. However, in the context of age, prior research has undertaken comparisons between distinct age cohorts, specifically young adults and older adults, in order to assess the impact of tES, whereas we have explored differences within a specific age category. Furthermore, there is no clear picture of increased efficacy in either age group. For example, our observations support the conclusions drawn by Peter et al.^87^, who showed that anodal tDCS over the left DLPFC can reduce stress and thus improve verbal episodic memory performance, but only in the young age cohort and not in older adults. On the other hand, our results contrast the findings of Antonenko et al.^84^, who demonstrated increased accuracy on an implicit language learning task following active theta tACS applied to the temporoparietal cortex only in old but not in young adults. Moreover, Fresnoza et al. found no differences between young and old adults in motor cortex excitability after tACS administered at the individual alpha frequency^86^, but strong evidence for higher effectiveness in older adults when targeting implicit motor learning^38^. Consequently, the presence of contradictory outcomes indicates potential interaction effects involving the specific age group, the employed tES methodology, and the cognitive domain under examination.

Regarding the influence of cognition on the efficacy of tES interventions, we can agree with the findings of de Sousa et al.^88^, who demonstrated enhanced training success following three sessions of combined object-location memory training and anodal tDCS (right temporoparietal cortex) only in MCI patients but not in healthy older adults. Another study conducted by Krebs et al.^89^ reported improved performance in a verbal episodic memory test after a single session of anodal tDCS over the left DLPFC, in the presence of a moderate degree of neuropathology, which is present in both high-education MCI patients and low-education AD patients, thus showing a three-way interaction. Instead of clinical diagnoses, the authors also examined whether a continuous variable for memory impairment (derived from the neurophysiological test battery CERAD) would yield disparate outcomes. However, their analysis revealed no significant divergence in results. In the context of CCT as a treatment tool to improve cognition, a meta-analysis showed strong evidence of efficacy in MCI patients, but only weak efficacy in the disease stage of dementia^90^. Despite the paucity of available studies examining an association between the effectiveness of tES intervention and the cognitive level, we hypothesize that, similar to the findings of Krebs et al.^89^ and Hill et al.^90^, interventions designed to improve specific cognitive skills have greater potential during the initial phases of cognitive decline as opposed to advanced stages.

In order to advance the exploration and enhancement of the impact of theta—gamma tACS on non-declarative memory functions, an integration of this technique with electrophysiological approaches, such as EEG^91^ or fMRI^92^, holds promise. This combined approach could facilitate the examination of either the immediate influence of stimulation on cerebral activity or connectivity, as well as the real-time adaptation of stimulation parameters based on ongoing brain activity (closed-loop). In addition, it would be conceivable to include an only tACS group in the study design in the future to be able to investigate the effect of controlling state-of-the-brain by CCT. The latter could possibly be enhanced by continuously adapting the difficulty level of the CCT to the individuals’ cognitive load. Moreover, in light of our discovered correlation indicating heightened efficacy of our intervention in individuals with lower baseline cognitive functioning, an exploration into the therapeutic potential in MCI patients holds significant scientific merit as it could have a positive impact on daily activities or quality of life. In general, fine-tuning or personalization of tACS parameters via electric field modeling based on individual head and brain anatomy could lead to reduced variability of treatment effects^93^. Further research is warranted to elucidate the distinctions between online and offline effects of tES interventions, as well as to assess the efficacy of multi-session tES protocols.

In conclusion, multi-session theta—gamma cross-frequency tACS is a well-tolerated tool with only mild AEs that effectively modulates non-declarative memory components in older adults by accelerating selectively their visuomotor learning not only in the short but also in the long-term. In contrast, the absence of any impact on probabilistic sequence-specific learning indicates a distinct dichotomy between these facets, signifying their reliance on discrete cerebral functions and structures. Our findings add to the literature by presenting empirical evidence that tACS can influence not only declarative but also non-declarative memory functions, with greater effectiveness in individuals of a younger age and those with pre-existing cognitive decline. The latter underscores the considerable scientific merit in exploring the therapeutic potential of our intervention in MCI patients as it could have a positive impact on daily activities or quality of life. However, it is imperative to investigate and optimize additional factors that influence the efficacy of this treatment to ultimately develop a comprehensive approach for enhancing implicit learning in individuals with age- or disease-related deficits.

### Supplementary Information

Supplementary Information.

## Data Availability

The raw data and the underlying code for data analysis and visualization are openly available in the ASRT_tACS repository and can be accessed via this link https://github.com/ludi94/ASRT_tACS.
