# Author Correction: Prefrontal theta—gamma transcranial alternating current stimulation improves non-declarative visuomotor learning in older adults

**DOI:** 10.1038/s41598-024-58939-2

**Published:** 2024-04-09

**Authors:** Lukas Diedrich, Hannah I. Kolhoff, Ivan Chakalov, Teodóra Vékony, Dezső Németh, Andrea Antal

**Affiliations:** 1 Department of Neurology, University Medical Center Göttingen, Göttingen, Germany; 2 Department of Anesthesiology, University Medical Center Göttingen, Göttingen, Germany; 3 Centre de Recherche en Neurosciences de Lyon CRNL U1028 UMR5292, INSERM, CNRS, Université Claude Bernard Lyon 1, Bron, France; 4 BML-NAP Research Group, Institute of Psychology, Eötvös Loránd University and Institute of Cognitive Neuroscience and Psychology, Research Centre for Natural Sciences, Budapest, Hungary; 5 Department of Education and Psychology, Faculty of Social Sciences, University of Atlántico Medio, Las Palmas de Gran Canaria, Spain

Correction to: *Scientific Reports* 10.1038/s41598-024-55125-2, published online 29 February 2024

The original version of this Article contained errors in Figure 4. In the legends for panels **a** and **b** the ‘ ≤ ’ and ‘ ≥ ’ signs did not display correctly.

The original Figure 4 and accompanying legend appear below.

**Figure 4:**
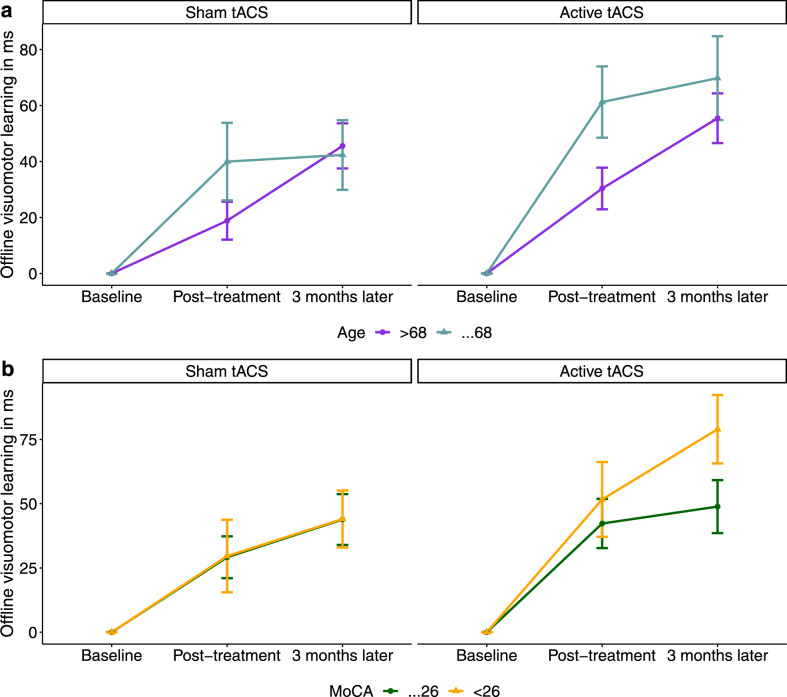
Active tACS treatment is more effective in younger and cognitively weaker participants. (**a**) In the active tACS group (upper right plot), older adults of younger age (≤ 68 years, light blue line, n = 18) revealed higher offline visuomotor learning than those of older age (> 68 years, purple line, n = 17) 3 months after treatment completion, whereas in the sham tACS group (upper left plot), both age groups showed the same level of offline visuomotor learning 3 months after treatment. (**b**) In the active tACS group (lower right plot), participants with MoCA (i.e. Montreal Cognitive Assessment) scores in the range of Mild Cognitive Impairment (MCI) (< 26, orange line, n = 17) revealed higher offline visuomotor learning than participants with MoCA scores in the healthy range (≥ 26, dark green line, n = 18) 3 months after treatment completion, whereas in the sham tACS group (lower left plot), both cognitively healthy and cognitively impaired participants exhibited the same level of offline visuomotor learning 3 months after treatment. The error bars represent the standard error of the mean (SEM).

The original Article has been corrected.

