# Supplementary material for: Prefrontal theta—gamma transcranial alternating current stimulation improves non-declarative visuomotor learning in older adults
Source: Sci Rep. 2024 Feb 29;14:4955. doi: 10.1038/s41598-024-55125-2 (PMC10901881; doi:10.1038/s41598-024-55125-2)
Supplement: Supplementary file 1 — Supplementary Information. [file 41598_2024_55125_MOESM1_ESM.pdf]

Supplementary Materials for:

Title: Prefrontal Theta—Gamma Transcranial Alternating Current Stimulation Improves Non-Declarative Visuomotor Learning in Older Adults

Authors: Lukas Diedrich[1], Hannah I. Kolhoff[1], Ivan Chakalov[1,2], Teodóra Vékony[3], Dezso Nemeth[3,4,†], Andrea Antal[1,†]

[1]Department of Neurology, University Medical Center Göttingen, Göttingen, Germany
[2]Department of Anesthesiology, University Medical Center Göttingen, Göttingen, Germany
[3]Université Claude Bernard Lyon 1, CNRS, INSERM, Centre de Recherche en Neurosciences de Lyon CRNL U1028 UMR5292, Bron, France
[4]BML-NAP Research Group, Institute of Psychology, Eötvös Loránd University & Institute of Cognitive Neuroscience and Psychology, Research Centre for Natural Sciences, Budapest, Hungary

† Senior authors

S1. Further methodological details

Reason	Dropouts
Illness	2
Covid infection	1
Covid anxiety	2
Personal reasons	1
No further interest	4
**Total**	**10**

Table S1.1: Dropout details. The numbers provided indicate the count of subjects who were unable to participate in the study due to the specified reasons.

	Sham tACS (n = 18)	Active tACS (n = 17)	p-value
Skin irritations	8 (3%)	2 (<1%)	0.28
Headache	2 (<1%)	5 (2%)	0.27
Vertigo	0	0	
Tiredness	4 (1%)	2 (<1%)	0.50
Nervousness	0	0	
Tingling	225 (78%)	193 (71%)	0.35
Phosphenes	7 (2%)	0	0.13

Table S1.2: Adverse effects and comparison between intervention groups. Numbers indicate the frequency with which an adverse effect occurred during stimulation sessions (e.g., *skin irritation* was reported 8 times in the sham-tACS group for a total of 288 sessions (18 participants times 16 treatment sessions)), a frequency of 3%. The column labeled "p-value" presents the observed distinctions between intervention groups. Two-sample two-sided t-tests were employed for statistical comparison. A p-value ≤ 0.05 indicates statistical significance.

S2. Full results of the linear mixed models

S2.1 Higher online visuomotor learning during baseline ASRT session

Note that this part complements the results presented in Section 3.1 in the main body of the paper. The full LMM results (Tab. S2.1) also revealed a difference in online (during practice sessions) visuomotor learning between sessions, which was indicated by a statistically significant and positive interaction effect of session [2] x epoch (beta = -0.76, 95% CI [-1.41, -0.10], $t(549) = -2.28$, $p = 0.023$; Std. beta = -0.05, Std. 95% CI [-0.10,-7.56e-03]) and a statistically significant and positive interaction effect of session [3] x epoch (beta = 8.94, 95% CI [5.03, 12.86], $t(826) = 4.48$, $p < .001$; Std. beta = 0.12, Std. 95% CI [0.07, 0.17]). Here, the highest amount of visuomotor learning was observed at baseline (Fig. S2.1).

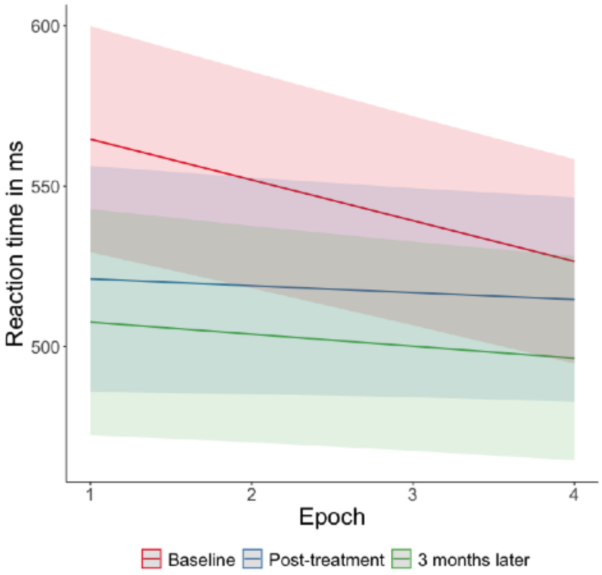

Figure S2.1: Higher online visuomotor learning during baseline ASRT session. Based on the LMM (Tab. S2.1), online visuomotor learning, meaning the decrease in reaction time across epochs, was more pronounced during the baseline session (red) of the Alternating Serial Reaction Time (ASRT) task compared to the learning during the post-treatment (blue) and follow-up (3 months later) (green) ASRT sessions. Solid lines represent regression fits and error bands denote 95% confidence intervals.

Predictor	N	Beta	95% CI[1]	Std. Beta	Std. 95% CI[1]	p-value
group						0.4
Sham tACS	432	—	—	—	—	
Active tACS	408	19	-27, 64	0.23	-0.30, 0.76	
session						
1	280	—	—	—	—	
2	280	-44	-52, -35	-0.33	-0.41, -0.26	**<0.001**
3	280	-57	-65, -49	-0.53	-0.60, -0.45	**<0.001**
trial type						**<0.001**
hpt	420	—	—	—	—	
lpt	420	7.3	3.8, 11	0.09	0.05, 0.13	
epoch	840	-13	-17, -8.9	-0.17	-0.22, -0.12	**<0.001**
group * session	840					
Active tACS * 2	136	-21	-30, -12	-0.25	-0.36, -0.15	**<0.001**
Active tACS * 3	136	-21	-29, -12	-0.25	-0.35, -0.14	**<0.001**
session * epoch	840					
2 * epoch	280	11	6.6, 14	0.14	0.09, 0.20	**<0.001**
3 * epoch	280	8.9	5.0, 13	0.12	0.07, 0.17	**<0.001**

[1]CI = Confidence Interval
Conditional $R^2 = 0.90$
Marginal $R^2 = 0.09$
AIC = 8119 (Estimated using *Maximum Likelihood*)

Table S2.1: Full results of the linear mixed model, predicting median RTs to investigate tACS treatment effects. The model's total explanatory power was substantial (conditional $R^2 = 0.90$) and the part related to the fixed effects alone (marginal R^2) was 0.09. The Akaike information criterion (AIC) of the model was estimated by the maximum likelihood method and was 8119. Standardized parameters were obtained by fitting the model on a standardized version of the dataset. 95% CIs and p-values were computed using a Wald t-distribution approximation. A p-value ≤ 0.05 indicates statistical significance (highlighted in bold).

S2.2 Higher online visuomotor learning during baseline session mediated by subjects with lower MoCA score

Note that this part complements the results presented in Section 3.2 in the main body of the paper. The full LMM results (Tab. S2.2) led to a specification of our previous findings (see findings shown in S.2.1) by showing that the differences in online (during practice sessions) visuomotor learning between sessions can only be observed for subjects in the MoCA „MCI-range“, mainly caused by the strong learning during the baseline session (Fig. S2.2). This was indicated by statistically significant and negative three-way interaction effects of (session [2] x MoCA) x epoch (beta = -2.81, 95% CI [-4.12, -1.50], t(814) = -4.21, p < .001; Std. beta = -0.11, 95% CI [-0.16,-0.06]) and (session [3] x MoCA) x epoch (beta = -2.17, 95% CI [-3.48, -0.87], t(814) = -3.26, p = 0.001; Std. beta = -0.08, Std. 95% CI [-0.14,-0.03]). Adding an epoch x session x age interaction term increased the AIC of the model (8061.7) while being non-significant for both session 2 and session 3 (p = 0.43; p = 0.35). Thus, the differences in online visuomotor learning between sessions were only affected by MoCA and not by age.

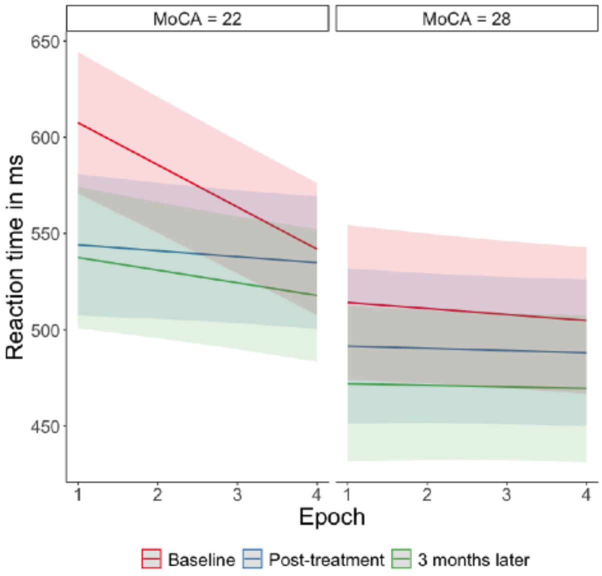

Figure S2.2: Higher online visuomotor learning during baseline session mediated by subjects with lower MoCA score. Based on the LMM (Tab. S2.2), subjects with lower cognition at baseline (left), as measured by the Montreal Cognitive Assessment (MoCA), exhibited differences in online visuomotor learning (decrease in reaction time (RT) across epochs) between Alternating Serial Reaction Time (ASRT) task sessions (Baseline - red; Post-treatment - blue; 3 months later - green). Participants with higher baseline cognition (right) showed similar online visuomotor learning

between sessions. Solid lines represent regression fits and error bands denote 95% confidence intervals.

Predictor	N	Beta	95% CI[1]	Std. Beta	Std. 95% CI[1]	p-value
group						0.15
Sham tACS	432	—	—	—	—	
Active tACS	408	27	-11, 65	0.33	-0.11, 0.77	
session						
1	280	—	—	—	—	
2	280	-43	-52, -35	-0.33	-0.40, -0.26	**<0.001**
3	280	-56	-65, -48	-0.52	-0.59, -0.45	**<0.001**
MoCA	840	-15	-25, -6.5	-0.38	-0.68, -0.08	**<0.001**
trial type						**<0.001**
hpt	420	—	—	—	—	
lpt	420	7.3	3.9, 11	0.09	0.05, 0.13	
age	840	2.6	-1.22, 6.4	0.21	-0.1, 0.51	0.183
epoch	840	-13	-16, -9.2	-0.17	-0.22, -0.13	**<0.001**
group * session	840					
Active tACS * 2	136	-20	-29, -12	-0.25	-0.35, -0.14	**<0.001**
Active tACS * 3	136	-22	-30, -13	-0.27	-0.37, -0.16	**<0.001**
group * MoCA	840					0.6
Active tACS * MoCA	408	-4.2	-19, 11	-0.15	-0.64, 0.35	
session * MoCA	840					
2 * MoCA	280	6.8	4.0, 9.6	0.09	0.02, 0.16	**<0.001**
3 * MoCA	280	4.6	1.9, 7.4	0.05	-0.02, 0.12	**0.001**
session * age	840					
2 * age	280	1.1	0.52, 2.3	0.11	0.04, 0.18	**0.002**
3 * age	280	-0.58	-1.5, 0.3	-0.05	-0.12, 0.02	0.199
group * age	840					0.5
Active tACS * age	408	2.0	-4.2, 8.2	0.16	-0.32, 0.65	
session * epoch	840					
2 * epoch	280	11	6.8, 14	0.14	0.09, 0.19	**<0.001**
3 * epoch	280	8.9	5.2, 13	0.12	0.07, 0.17	**<0.001**
MoCA * epoch	840	3.1	1.9, 4.3	0.12	0.08, 0.17	**<0.001**
group * session * MoCA	840					
Active tACS * 2 * MoCA	136	-2.3	-5.5, 0.99	-0.08	-0.19, 0.03	0.172
Active tACS * 3 * MoCA	136	3.3	-0.01, 6.5	0.11	0.0, 0.23	0.051
group * session * age	840			0.011		
Active tACS * 2 * age	136	0.23	-1.2, 1.6	0.02	-0.09, 0.13	0.743
Active tACS * 3 * age	136	1.9	0.55, 3.3	0.16	0.04, 0.27	**0.006**
session * MoCA * epoch	840					
2 * MoCA * epoch	280	-2.8	-4.1, -1.5	-0.11	-0.16, -0.06	**<0.001**
3 * MoCA * epoch	280	-2.2	-3.5, -0.87	-0.08	-0.14, -0.03	**0.001**

[1]CI = Confidence Interval
Conditional $R^2 = 0.91$
Marginal $R^2 = 0.47$
AIC = 8051 (Estimated using *Maximum Likelihood*)

Table S2.2: Full results of the linear mixed model, predicting median RTs to investigate correlations between tACS treatment efficacy, age and baseline cognition. The model's total explanatory power was substantial (conditional $R^2 = 0.91$) and the part related to the fixed effects alone (marginal R^2) was 0.47. The Akaike information criterion (AIC) of the model was estimated by the maximum likelihood method and was 8051. Standardized parameters were obtained by

fitting the model on a standardized version of the dataset. 95% CIs and p-values were computed using a Wald t-distribution approximation. A p-value ≤ 0.05 indicates statistical significance (highlighted in bold).

S3. Further analyses

S3.1 How is implicit learning affected by age and cognition (baseline data only) ?

To explore correlations between implicit learning and various aspects of aging (cognition, age), we fitted a LMM to predict RT with epoch (1-4), trial type (hpt vs. lpt), MoCA (1-30) and age (55-85) (formula: RT ~ epoch * MoCA + trial type * age) (Tab. S3.1). The model included a random intercept for subject and a by-subject random slope for the effect of epoch (formula: ~1 + epoch | subject). The model's total explanatory power is substantial (conditional R^2 = 0.95) and the part related to the fixed effects alone (marginal R^2) is of 0.34 (AIC = 2695).

The model revealed significant *sequence-specific learning* such that subjects were faster on hpt than on lpt, which was indicated by a statistically significant and positive effect of trial type [lpt] (beta = 6.71, 95% CI [2.05, 11.37], t(269) = 2.84, p = 0.005; Std. beta = 0.07, Std. 95% CI [0.02, 0.13]). This sequence-specific learning effect decreases with increasing age, which was shown by a statistically significant and negative interaction effect of trial type [lpt] x age (beta = -0.79, 95% CI [-1.49, -0.10], t(269) = -2.24, p = 0.026; Std. beta = -0.06, Std. 95% CI [-0.11,-0.01]) (Fig. S3.1a). Adding a trial type x MoCA interaction term increased the AIC of the model (2697.1) while being non-significant (p = 0.781). Thus, there was no significant effect of the individual MoCA score on sequence-specific learning.

The model also revealed significant *visuomotor learning* such that RT decreased across epochs, which was indicated by a statistically significant and negative effect of epoch (beta = -12.70, 95% CI [-17.56, -7.83], t(269) = -5.14, p < .001; Std. beta = -0.16, Std. 95% CI [-0.22, -0.10]). This visuomotor learning effect correlates negatively with increasing individual MoCA score, which was shown by a statistically significant and positive interaction effect of epoch x MoCA (beta = 3.13, 95% CI [1.45, 4.82], t(269) = 3.66, p < .001; Std. beta = 0.11, Std. 95% CI [0.05, 0.17]) (Fig. S3.1b). Adding an epoch x age interaction term increased the AIC of the model (2697.1) while being non-significant (p = 0.757). Thus, there was no significant effect of age on visuomotor learning.

Generally, both increasing age and increasing cognitive impairment led to slower RT's, which was indicated by a statistically significant and negative effect of MoCA (beta = -17.06, 95% CI [-26.97, -7.14], t(269) = -3.39, $p < .001$; Std. beta = -0.40, Std. 95% CI [-0.67, -0.12]) and a statistically significant and positive effect of age (beta = 4.38, 95% CI [1.05, 7.72], t(269) = 2.59, p = 0.010; Std. beta = 0.33, Std. 95% CI [0.08, 0.58]).

Our findings are in line with the results of Janacsek et al. (2012) and Juhasz et al. (2019) by demonstrating a decrease in probabilistic sequence-specific learning with age. By integrating individual cognitive level as a continuous variable, we extend the work of Nemeth et al. (2013), who compared MCI patients to controls in terms of their probabilistic sequence-specific learning performance and found lower learning performance in the former. Unexpected in view of the results of Nemeth et al., we did not find a correlation between sequence-specific learning and the individual cognitive level. The lack of correlation may be due to the fact that in our study only the MoCA test was applied to determine the subjects' cognitive level, whereas the subjects in the study of Nemeth et al. underwent a full MCI diagnosis. Contrary to sequence-specific learning, visuomotor learning was unaffected by age but more pronounced in cognitively impaired older adults. Regarding the influence of age on visuomotor learning, a former lifespan study by Juhasz et al. (2019) found age-related differences indicating higher learning in children and older adults compared to adolescents and young adults. Therefore, we assume that our age range under investigation (55-82 years) was not wide enough to reveal any differences. To conclude, different aspects of implicit learning are differently affected by various aspects of aging. Probabilistic sequence-specific learning declines with age and remains unaffected by initial cognitive decline. Visuomotor learning does not exhibit significant variations within the age range under investigation (55-82 years). However, it does demonstrate "more room to improve" in individuals with lower cognitive abilities.

Predictor	N	Beta	95% CI[1]	Std. Beta	Std. 95% CI[1]	p-value
epoch	280	-13	-18, -7.7	-0.16	-0.22, -0.10	**<0.001**
MoCA	280	-17	-27, -6.8	-0.40	-0.67, -0.12	**<0.001**
tripletType						**0.005**
H	140	—	—			
L	140	6.7	2.0, 11	0.07	0.02, 0.13	
age	280	4.4	0.93, 7.8	0.33	0.08, 0.58	**0.010**
epoch * MoCA	280	3.1	1.4, 4.9	0.11	0.05, 0.17	**<0.001**
tripletType * age	280					**0.025**
L * age	140	-0.79	-1.5, -0.10	-0.06	-0.11, -0.01	

[1]CI = Confidence Interval
Conditional $R^2 = 0.95$
Marginal $R^2 = 0.34$
AIC = 2673

Table S3.1: Results of the linear mixed model, predicting median RTs to investigate correlations between implicit learning, age and cognition (baseline data only). The model's total explanatory power was substantial (conditional $R^2 = 0.95$) and the part related to the fixed effects alone (marginal R^2) was 0.34. The Akaike information criterion (AIC) of the model was estimated by the maximum likelihood method and was 2673. Standardized parameters were obtained by fitting the model on a standardized version of the dataset. 95% CIs and p-values were computed using a Wald t-distribution approximation. A p-value ≤ 0.05 indicates statistical significance (highlighted in bold).

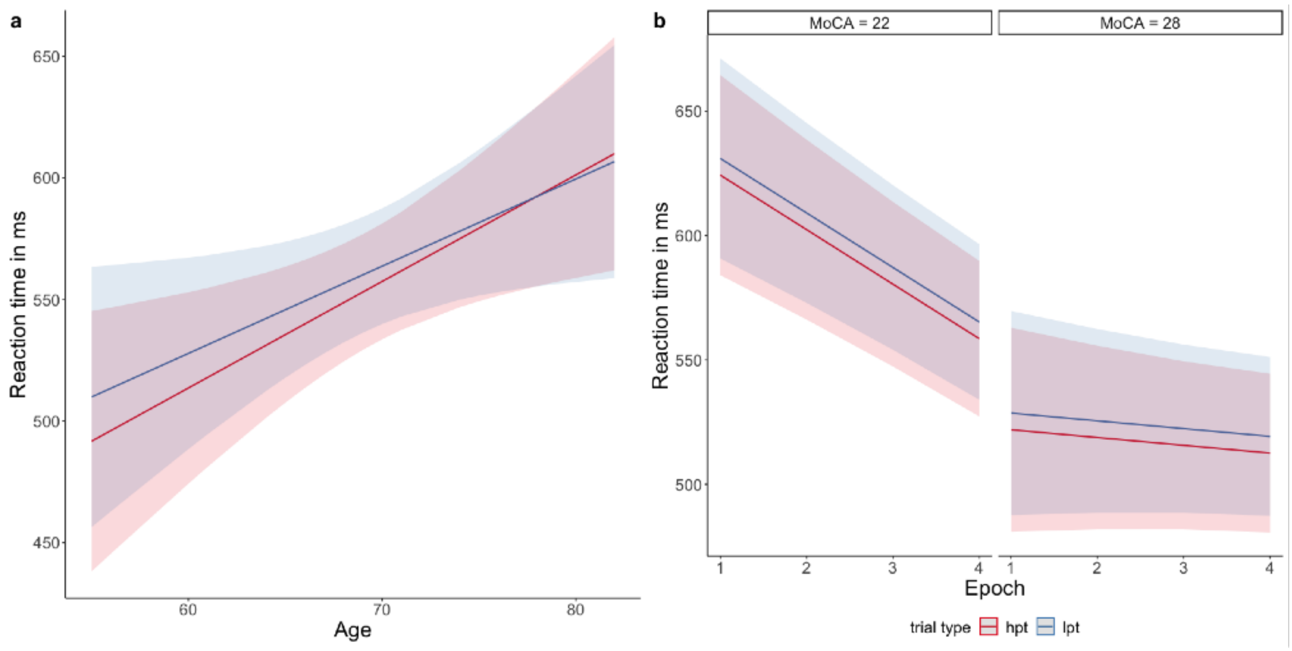

Figure S3.1: Baseline age and cognition affect aspects of implicit learning differently. A linear mixed model based on the baseline data (Tab. S3.1) showed a) a decrease of sequence-specific learning, meaning the difference in reaction time (RT) between high-probability triplets (hpt, red) and low-probability triplets (lpt, blue), with increasing age, as well as b) a larger visuomotor learning effect, meaning the decrease in RT across epochs, for subjects with lower baseline cognition, as measured by the Montreal Cognitive Assessment (MoCA). Solid lines represent regression fits and error bands denote 95% confidence intervals.

References

Janacsek K, Fiser J, Nemeth D (2012): The best time to acquire new skills: age-related differences in implicit sequence learning across the human lifespan: Implicit learning across human lifespan. Developmental Science 15, 496–505

Juhasz, D., Nemeth, D., & Janacsek, K. (2019). Is there more room to improve? The lifespan trajectory of procedural learning and its relationship to the between- and within-group differences in average response times. *PloS one*, *14*(7), e0215116. https://doi.org/10.1371/journal.pone.0215116

Nemeth D, Janacsek K, Fiser J (2013): Age-dependent and coordinated shift in performance between implicit and explicit skill learning. Front Comput Neurosci 7
